# Efficient biodegradation of di-(2-ethylhexyl) phthalate by a novel strain *Nocardia asteroides* LMB-7 isolated from electronic waste soil

**DOI:** 10.1038/s41598-022-19752-x

**Published:** 2022-09-10

**Authors:** Tian-Tian Chang, Zhi-Wei Lin, Liu-Qing Zhang, Wei-Bing Liu, Ying Zhou, Bang-Ce Ye

**Affiliations:** Lab of Biosystems and Microanalysis, State Key Laboratory of Bioreactor Engineering, East China University of Science and Technology, Meilong RD 130, Shanghai, 200237 China

**Keywords:** Applied microbiology, Environmental microbiology, Environmental biotechnology

## Abstract

The di-2-ethylhexyl phthalate (DEHP) degrading strain LMB-7 was isolated from electronic waste soil. According to its biophysical/biochemical characteristics and 16S rRNA gene analysis, the strain was identified as *Nocardia asteroides*. Optimal pH and temperature for DEHP degradation were 8.0 and 30 °C, respectively, and DEHP removal reached 97.11% after cultivation for 24 h at an initial concentration of 400 mg/L. As degradation intermediates, di-butyl phthalates, mono-2-ethylhexyl phthalate and 2-ethylhexanol could be identified, and it could be confirmed that DEHP was completely degraded by strain LMB-7. To our knowledge, this is a new report of DEHP degradation by a strain of *Nocardia asteroides*, at rates higher than those reported to date*.* This finding provides a new way for DEHP elimination from environment.

## Introduction

Phthalic acid esters (PAEs) are a class of refractory organic compounds predominantly used as plasticizers to improve mechanical properties (flexibility, extensibility and durability) in plastic products. Flexible plastic products such as food packaging materials, household products, and medical supplies are widely used in our daily life. Phthalates are considered as weakly estrogenic environmental contaminants. Jobling et al.initial concentration

^1^ indicated that neither benzyl butyl phthalate (BBP) nor di-n-butyl phthalate (DBP) was able to act as antagonists in the presence of endogenous estrogens, and that their overall effect would be cumulative. Among various phthalate esters, di-(2-ethylhexyl) phthalate (DEHP) is one of the most widely used phthalates in China, for it serves as an important additive to make polyvinyl chloride (PVC) soft and flexible. However, DEHP is easily released into the environment, and consumer products containing DEHP can result in human exposure through direct contact and use. Exposure to high concentrations of DEHP was shown to produce a wide range of adverse effects in experimental animals, including cancer, liver damage, birth defects, and alterations of the reproductive system, especially for male animals^2,3^. It has been reported that the application of plastic mulch film for the cultivation of vegetables led to DEHP being taken up by plants and entering the human food chain^4,5^.

In nature, phthalates are environmental contaminants which cannot be degraded easily. The degradation of DEHP by microorganisms is the most effective means of removing it from aquatic and terrestrial systems, and is the most promising process for remediating contaminated environments, especially for greenhouse soils. A large number of different microorganisms with DEHP-degrading abilities have been reported previously, belonging to *Microbacterium* sp. *Agromyces* sp. and *Gordonia alkanivorans*, *Rhodococcus pyridinivorans*^6–9^. However, most of these microorganisms cannot use DEHP as the sole carbon source. While many microorganisms have been described to be able to degrade phthalates, degradation rates decreased with increasing alkyl chain length and presence of alkyl branch chains^10^. DEHP with its long side chain is among the most difficult to be utilized phthalates^11^.

Members of the genus *Nocardia* are widely distributed in soils. There are few reports on *Nocardia* spp. able to degrade environmental pollutants, including several reports on their ability to degrade crude oil^12–14^. Furthermore, Shetty and Shetty^15^ identified the pathway for the degradation of phenol by *Nocardia hydrocarbonoxydans* NCIM 2386, and Hristov et al*.* showed that a *Nocardia* sp. could mineralize phenol efficiently^16^. However, to date there was one report on the potential for biodegradation of phthalates in *Nocardia* spp.^17^.

In this study, a novel bacterial strain LMB-7 that could degrade DEHP efficiently was isolated and characterized. The optimum conditions of DEHP degradation were determined, and degradation kinetics were investigated in liquid medium. Furthermore, the pathway of DEHP degradation was elucidated based on the intermediate metabolites identified by gas chromatography mass spectrometry (GC–MS). Therefore, the LMB-7 strain isolated in this work could be used to remove DEHP from environment, which provides a new tool for environmental governance.

## Materials and methods

### Chemicals and culture media

The PAEs used in this study, including di-methyl phthalate (DMP), di-ethyl phthalate (DEP), di-butyl phthalate (DBP) and DEHP, were purchased from Sigma-Aldrich Chemie GmbH (Hamburg, Germany), and purities were all above 99.5%. Methanol with HPLC grade was purchased from Solarbio (Beijing, China). All other chemical reagents were also analytical grade, and all solvents were HPLC grade.

In this study (enrichment and degradation experiment), a mineral salts medium (MSM) was used to culture the cells in liquid culture. MSM contained, per litre: 1 g of NaCl, 1.5 g of K_2_HPO_4_, 0.75 g of KH_2_PO_4_, 1 g of NH_4_NO_3_ and 0.2 g of MgCl_2_. The pH of the medium was adjusted to 7.0 with HCl or NaOH. DEHP was added to the medium as the sole carbon source. All of the media were sterilized by autoclaving for 20 min at 121 °C.

### Enrichment and isolation of DEHP-degrading bacteria

In this study, six soil samples from an electronics factory were collected and stored at 4 °C for one week prior to mixing and use. DEHP degrading bacteria were enriched using a procedure described previously^18^. Briefly, 5 g of mixed soil sample was added to 100 mL MSM with DEHP (100 mg/L) as the sole carbon source and incubated for 7 days at 30 °C and in a shaker (220 rpm). After that, 2 mL suspension was removed and added to fresh 100 mL MSM with DEHP (200 mg/L). After 7 days of culture at 30 °C and 220 rpm, 2 mL of the suspension was added to 100 mL of fresh MSM containing 300 mg/L DEHP. After three rounds of enrichment culture, the final culture was obtained. Then, 0.1 mL of the culture was evenly spread onto an MSM plate containing 2.0% agar with DEHP (200 mg/L) as the sole carbon source. Nine colonies were re-spread on MSM agar plates with DEHP (200 mg/L) as the sole carbon source to confirm purity and ability to use DEHP. Then, one clone which grew fast on MSM solid medium containing DEHP was picked and inoculated into liquid MSM medium. The bacteria were cultured for 24 h before stored with 25% (v/v) glycerol at −20 °C for further study.

### 16S rRNA gene sequence analysis

Through screening, LMB-7 was considered to have strong DEHP degradation capacity and was phylogenetically characterized using 16S ribosomal RNA (rRNA) gene sequencing. Genomic DNA of strain LMB-7 was extracted using the DNA Rapid Extraction Kit (TransGen Biotech, Beijing, China). Primers 27F: 5’-AGAGTTTGATCCTGGCTCAG-3’ and 1492R: 5’-GGCTTCCTTGTTACGACTT-3’ were used to amplify full-length 16S rRNA genes, and PCR products were sequenced by Major Bio Corporation (Shanghai, China). The resulting near-full length 16S rRNA gene sequence of LMB-7 (1425 bp) was deposited in the GenBank database with accession number MH734910. Furthermore, the sequence was compared to known bacterial sequences using the Basic Local Alignment Search Tool.

### Biodegradation tests of DEHP

LMB-7 was inoculated into 30 mL MSM medium with DEHP (200 mg/L) as the sole carbon source and cultured at 30 °C, 220 rpm for 24 h as a seed for degrading experiments. Subsequent cultures (30 mL) were inoculated with 1.0% of this pre-culture (v/v), unless stated otherwise. The substrate concentration in the medium was 400 mg/L. All the cultures were incubated at 30 °C and 220 rpm for 24 h. After 24 h, the remaining substrate in the medium was extracted and detected as described below to determine the extent of substrate degradation.

Growth of LMB-7 was determined in MSM medium with DEHP (200 mg/L) as the sole carbon source. Absorption at 600 nm was measured spectrophotometrically every 4 h and up to 24 h.

For different initial concentrations of DEHP (100, 200, 400 and 600 mg/L), the degradation rate of DEHP by LMB-7 was determined. Degradation curves were fitted for all concentrations and drawn with first-order kinetics that is frequently used to describe the biodegradation of organic matter^19^.

The formula of this equation is

1 $${\text{LnC }} = - {\text{Kt }} + {\text{A}}$$

where C is the DEHP initial concentration, K is the first-order kinetic constant, t is time, and A is the constant. The half-life of DEHP degradation was calculated as

2 $${\text{T}}_{{{1}/{2}}} = {\text{ln2}}/{\text{K}}$$

### Analytical methods

DEHP and its metabolites were extracted from the cell-free filtrates using solid phase extraction (SPE)^20^. Briefly, cultures (30 mL) were centrifuged at 8000×*g* for 10 min at 25 °C, and the supernatant was adjusted to pH 6.0 with 2.0 M HCl. The SPE column (Supelco/Sigma-Aldrich, Bellefonte, PA) was activated with 2 mL methanol first and balanced with 2 mL deionized water. Then, samples were passed through the SPE column as slowly as possible (approximately 1 mL/min). After passing the complete sample, the impurities on the column were washed with 2 mL of deionized water. Finally, DEHP was eluted with methanol (2 mL) from the column and all samples were passed through a 0.22 μm membrane filter before HPLC analysis.

The concentration of DEHP in the sample was determined by HPLC^21^. The chromatographic separation was performed on a C18 column (250 × 4.6 mm I.D., 5 μm; Teknokroma). The mobile phase for detecting DEHP was methanol: water (90: 10, v/v), the flow rate was 1 mL/min, and UV detection was done at 225 nm.

The metabolic products of DEHP in the fermentation liquid of strain LMB-7 containing DEHP (200 mg/L) were extracted and identified by gas chromatography–mass spectrometry (GC–MS) (Agilent 6890 Gas Chromatography-5975I Mass, Anaheim, CA). The injector temperature was set at 250 °C. The interface and ion source temperature were both set to 280 °C. The column temperature was increased from 100 °C for 1 min, raised at 10 °C /min to 200 °C, then at 6 °C /min to 280 °C (3 min hold). The culture conditions were the conditions for the highest degradation rate of DEHP. The same culture supernatants lacking LMB-7 were used as a negative control.

### Ethics approval and consent to participate

This article does not contain any studies with human participants or animals performed by any of the authors.

## Results

### Isolation and identification of strain LMB-7

After enrichment and selection with increasing concentrations of DEHP, an isolate with DEHP degrading ability was obtained from soil polluted with electronic waste (Lishui of Jiangsu province, China) and designated as LMB-7. Colonies of strain LMB-7 grown on MSM agar for two days were circular, with untidy edges, opaque, convex and yellowish, and with lack of aerial mycelia (Fig. S1A). The cells were rod-shaped and stained Gram-positive (Fig. S1B). Phylogenetic analysis of the 16S rRNA gene sequence revealed that strain LMB-7 is grouping with members of the genus *Nocardia*, being most closely related to *Nocardia asteroides* SB15 (GenBank accession number KC577155) with the 16S rRNA genes of both strains being identical.

### Cell growth

LMB-7 strain grew well in MSM medium with 100 mg/L DEHP as the sole carbon source. The maximum OD_600_ was 0.20 ± 0.02 after 24 h of incubation (Fig. S2. Meanwhile strain LMB-7 was prone to stick to the wall of the glass cultivation flask when growing in MSM medium.

### Substrate utilization tests

**Figure 1:**
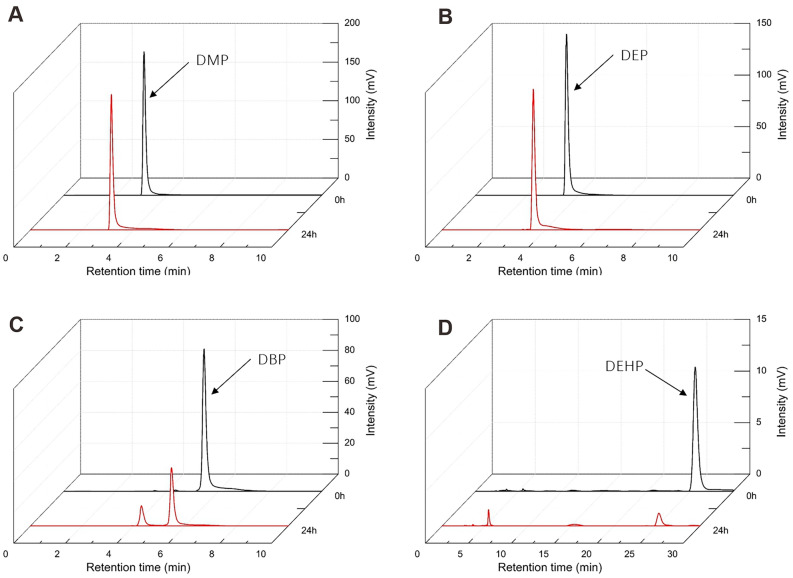
HPLC elution profiles (red lines) of substrate and metabolites during growth of strain LMB-7, using (**A**) DMP, (**B**) DEP, (**C**) DBP and (**D**) DEHP as the sole carbon source, respectively, after 24 h of incubation. Black lines are from reference standards of single compounds.

We then proceeded with analyzing the substrate profile as DEHP-contaminated environments are often characterized by the co-occurrence of several types of PAEs and other organic compounds^22,23^. We tested the ability of LMB-7 to degrade four different PAEs (DMP, DEP, DBP and DEHP) at an initial concentration of 400 mg/L and cultivation at 30 °C, 220 rpm, and pH 7.0 for 24 h. These four different PAEs were chosen based on the different length of the side chain. After 24 h of cultivation, 2.69% DMP, 6.17% DEP, 63.76% DBP and 87.04% of DEHP were removed (Fig. 1).

### Optimization of culture conditions for DEHP degradation by strain LMB-7

**Figure 2:**
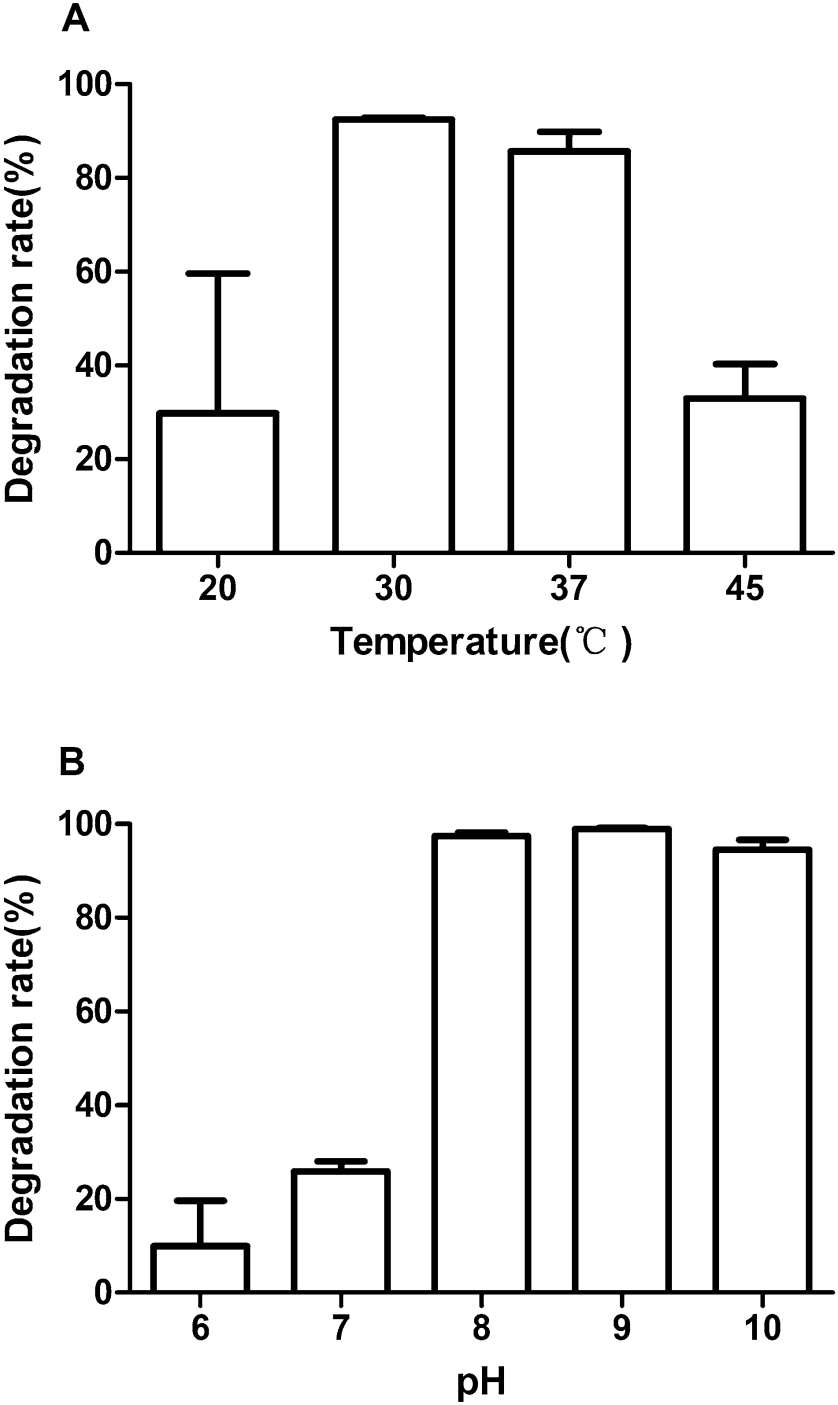
Extent of DEHP degradation by strain LMB-7 under different conditions for 24 h incubation. (**A**) Degradation of DEHP at different temperatures; (**B**) Degradation of DEHP at different pH. Data are the mean ± SD of three independent experiments.

Environmental factors such as pH, temperature and salinity play an important role in affecting the efficiency of biodegradation, including microbial growth and enzymatic activity. The DEHP degradation efficiency of strain LMB-7 was measured at temperatures ranging from 20 to 45 °C. DEHP degradation increased from 23.41% (20 °C) to 92.52% (30 °C) within 24 h at pH 7.0 when the concentration was 200 mg/L (Fig. 2A). The optimal temperature was around 30 °C. Furthermore, DEHP degradation by strain LMB-7 was tested at pH values ranging from 6.0 to 10.0. The degradation efficiency was over 97.50% at pH 8.0–10.0, and 200 mg/L DEHP was almost completely removed after 24 h (Fig. 2B). Compared with lower pH values, strain LMB-7 degraded DEHP faster at pH values of 8 and higher, meaning alkaline conditions are more suitable for DEHP degradation by LMB-7.

### Biodegradation of DEHP at different initial concentrations

**Figure 3:**
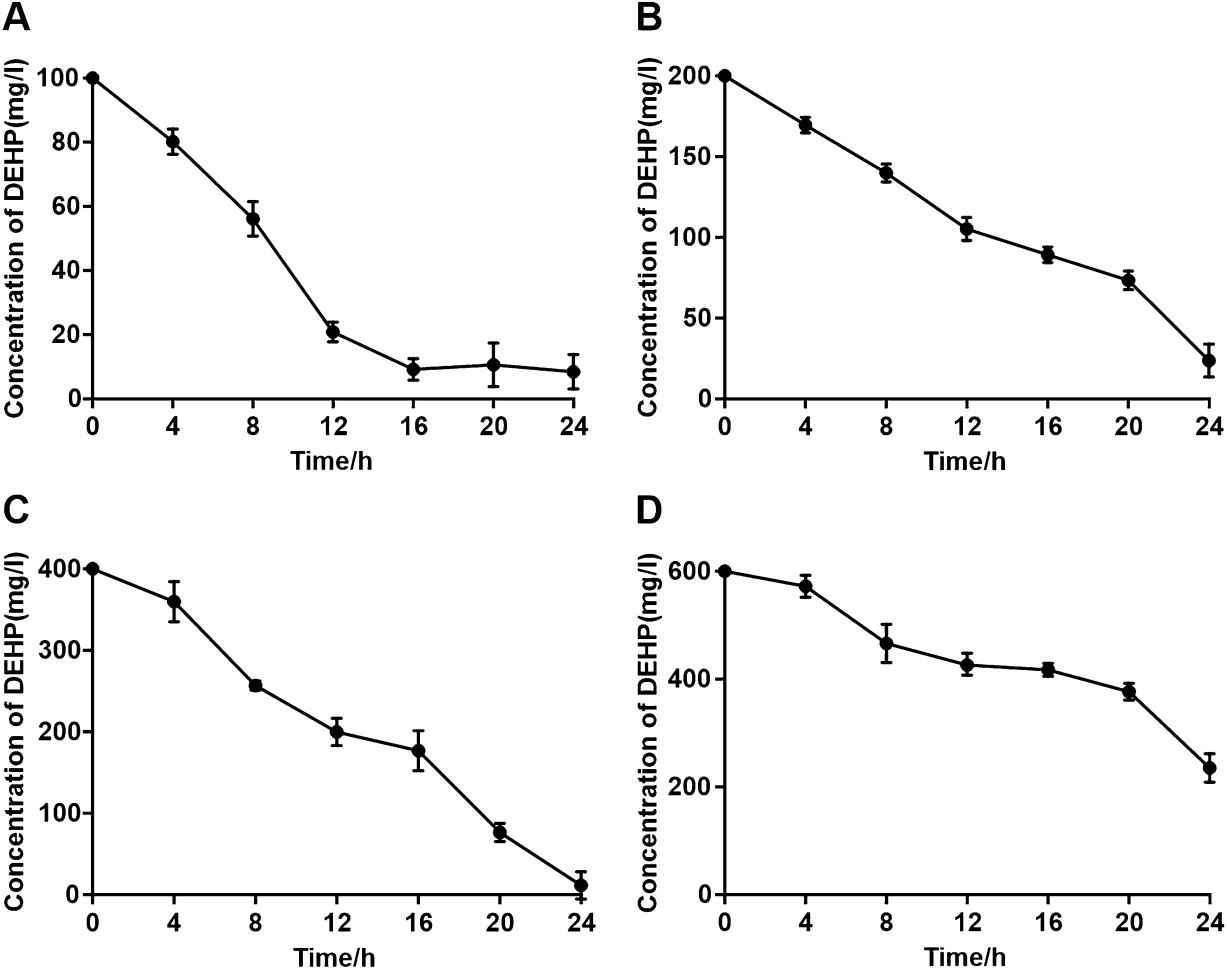
Degradation of DEHP by LMB-7 in MSM containing different initial DEHP concentrations. Initial concentrations of DEHP were 100, 200, 400 and 600 mg/L, respectively, for (**A**–**D**). Data are the mean ± SD of at least three independent experiments.

The biodegrading ability of *Nocardia asteroides* LMB-7 using different initial DEHP concentrations was also investigated in this study. The initial DEHP concentration ranged from 100 to 600 mg/L in MSM. After incubation for approximately 24 h, 91.55%, 88.14%, 97.11%, and 60.77% of total DEHP was degraded when the initial concentrations were 100, 200, 400 and 600 mg/L, respectively (Fig. 3). At 600 mg/L, degradation was slower than at lower concentrations. Using first-order kinetics, the fitness of the models was relatively high (> 0.9). The lowest degradation half-life found in this study was 6.43 h when the initial DEHP concentration was 100 mg/L (Table 1).

**Table 1:** Kinetics of DEHP degradation by *Nocardia asteroides* LMB-7 at different initial concentrations.

Initial concentration (mg/L)	Kinetic equations	Half-life (h)	R^2^
100	lnC = −0.1185t + 4.6740	6.43	0.9146
200	lnC = −0.0516t + 5.3188	13.83	0.9947
400	lnC = −0.0760t + 6.1333	10.98	0.9039
600	lnC = −0.0372t + 6.4701	20.59	0.9169

### Identification of DEHP degradation intermediates

**Figure 4:**
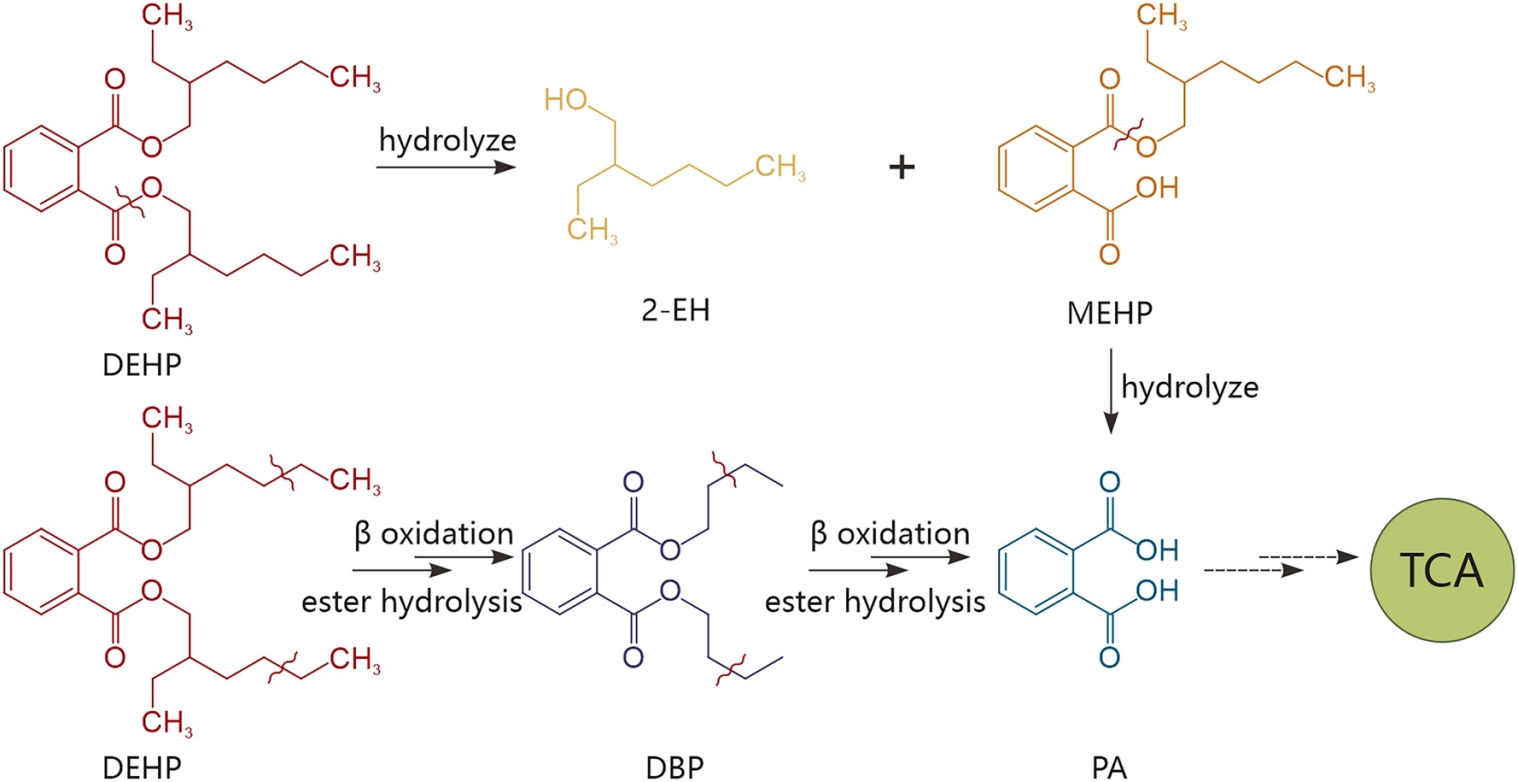
The proposed pathway of DEHP degradation by LMB-7 based on the presumed metabolites. 2-EH, 2-Ethylhexanol; PA, phthalic acid; TCA, tricarboxylic acid cycle.

Based on the GC–MS analysis and the corresponding chemical properties, the possible degradation pathway of DEHP by *Nocardia asteroides* LMB-7 was identified (Fig. 4). As shown in Fig. S3, three metabolites were detected by GC–MS, in which, the peaks of *m*/*z* 57, *m*/*z* 149, 163 and *m*/*z* 149 indicate the products of 2-ethylhexanol (2-EH), monoethylhexyl phthalate (MEHP) and DBP, respectively. All three peaks decreased gradually, and none of them were detected at the end of the cultivation (24 h), indicating that *Nocardia asteroides* LMB-7 could use DEHP and its metabolites as a sole carbon source. Thus, we can speculate that there may be two metabolic pathways for DEHP in LMB-7.

## Discussion

An efficient DEHP-degrading bacterium utilizing DEHP as sole carbon and energy source was isolated and characterized from an electronic waste dump site, and was designated as strain LMB-7. According to its 16S rRNA gene sequence, morphology, and physico-biochemical characteristics, strain LMB-7 belongs to *Nocardia asteroides*. To the best of our knowledge, this is a new reported case of DEHP degradation by a strain of *Nocardia asteroides*. Some studies have shown that *Nocardia* spp. can degrade rubber to reduce the degree of rubber contamination^24,25^. Metabolic breakdown of PAEs by microorganisms is considered to be one of the major and effective routes of environment bioremediation. It has been shown that PAEs with longer alkyl chains are more difficult to be degraded than those with shorter alkyl chains^26,27^. In contrast to this general assumption, degradation of PAEs by strain LMB-7 increased with increasing length of the alkyl chains (Fig. 1). This phenomenon should be studied in more detail in the future, addressing its biochemical and genetic basis.

Furthermore, degradation of DEHP by LMB-7 was very efficient. Disappearance of DEHP amounted to 98.9% under optimal conditions (pH 8.0 and 30 °C) at the end of 24 h. This is much faster than what has been reported before for other strains (Table 2), including some strains isolated by us previously^18,28^. Previously isolated strains were able to degrade both DBP and DEHP efficiently, but none of these could reach over 97% degradation at the end of 24 h. Among the different strains previously reported for DEHP degradation, except for two strains (*Sphigomonas* sp.DK4, *Corynebacterium* sp. O18^29^) with weaker degradation ability (less than 30%), the others can reach more than 94% (G. polyisoprenivorans G1^30^, Rhodococcus sp. strain WJ4^31^, Fusarium culmorum^32^, Agromyces sp. MT-O^8^, Rhodococcus ruber YC-YT1^33^, Gordonia alkanivorans YC-RL2^7^, Microbacterium sp. CQ0110Y^6^), but the fastest two of them takes 3 days, and the other strains take 6 days or more. Therefore, in comparison, LMB-7 is very efficient in degrading DEHP. Actually, the complex natural environment may affect the degradation ability of microorganisms. In the future, LMB-7 needs to be optimized to better adapt to different natural environments.

**Table 2:** Comparison of LMB-7 with other reported species regarding their ability to degrade DEHP.

Strain	Original source	Condition	Degradation	References
*Sphigomonas* sp.DK4	River sediment	30 ℃, pH 7	7 days, 100 mg L^−1^ 21.5%	^29^
*Corynebacterium* sp. O18	Petrochemical sludge	30 ℃, pH 7	7 days, 100 mg L^−1^ 21.2%	
*G. polyisoprenivorans* G1	Soil sample	In aqueous medium	3 days, 100 mg L^−1^ 97%	^30^
*Rhodococcus* sp. strain WJ4	Soil sample	28 ℃, in the dark	7 days, 200 mg L^−1^ 96.4%	^31^
*Fusarium culmorum*	Mixed pulper wasters	28 ℃, pH 6.5	6 days, 1000 mg L^−1^ with Glucose,98%	^32^
*Agromyces* sp. MT-O	Soil sample	29.6 ℃, pH 7.2	7 days, 200 mg L^−1^ 100%	^8^
*Rhodococcus ruber* YC-YT1	Marine plastic debris	30 ℃, pH 7.0	3 days, 100 mg L^−1^ 100%	^33^
*Gordonia alkanivorans* YC-RL2	Petroleum-contaminated soil	30 ℃, pH 8.0	7 days, 800 mg L^−1^ 94.6%	^7^
*Microbacterium* sp. CQ0110Y	Activated sludge	30 ℃, pH 7.0	10 days, 1350 mg L^−1^ 100%	^6^
*Nocardia asteroides* LMB-7	Electronic waste soil	30 ℃, pH 8.0	24 h, 200 mg L^−1^ 98.9%	This study

In the kinetic analysis, we selected a first-order kinetic model with a substrate inhibition coefficient to simulate DEHP degradation. DEHP biodegradation data fitted well with the exponential model, lnC = −Kt + A, confirming that degradation proceeded following first-order kinetics when DEHP was the sole carbon source.

Metabolite analyses confirmed the occurrence of MEHP, DBP and 2-EH during the degradation process (Fig. S3). As most metabolite pathway of phthalates has been reported, the first step is hydrolysis of the ester side-chain of the di-alkyl phthalate to generate mono-alkyl phthalate, and the other ester side-chain is broken to generate PA and alkyl alcohols available for further conversion. However, according to the proposed degradation pathways of DEHP by strain LMB-7 (Fig. 4), DEHP was hydrolyzed to MEHP and DBP catalyzed by esterase and β-oxidase simultaneously, then both MEHP and DBP were catalyzed by esterase to produce PA^32^. PA is the central intermediate of DEHP biodegradation, and the utilization of PA contribute to the complete degradation of DEHP. Several lines of evidences demonstrate that PA is usually utilized through aerobic and anaerobic pathways, which have been systematically introduced by Liang et al.^34^. In the aerobic pathway, PA is transformed to protocatechuate^35,36^ through *cis*-4,5-dihydro-4,5-dihydroxyphthalate in Gram-negative bacterium or *cis*-3,4-dihydro-3,4-dihydroxyphthalate and 3,4-dihydroxyphthalate in Gram-positive bacterium^35^. Following protocatechuate is degraded to pyruvate and oxaloacetate through 4-carboxy-2-hydroxymuconic semialdehyde, 2-hydroxy-4-carboxymuconic semialdehyde-hemiacetal, and 4-oxalocitramalate^35,37^. In the anaerobic pathway, PA is metabolized to benzoate by decarboxylation^38,39^, which is then transformed to acetate by β-oxidation^40^. Future experiments using isotope-labelled substrates can contribute to further elucidate and confirm the detailed degradation pathway in LMB-7.

## Conclusion

In this study, a DEHP degrading strain, *Nocardia asteroides* LMB-7, was isolated from electronic waste soil. It could remove 400 mg/L DEHP within 24 h. This is a new reported example of DEHP degradation by *Nocardia asteroides*. Future studies should address the enzymes in this strain involved in DEHP degradation, as degradation was faster and more complete than that of most other reported strains.

## Supplementary Information

Supplementary Information.

## Data Availability

Gene sequences used in compared are from Genbank (http://www.ncbi.nlm.nih.gov/) and the material and data supporting their findings can be found in the main paper and the supporting materials (https://www.ncbi.nlm.nih.gov/nuccore/MH734910, Accession Number MH734910).

## Abbreviations

DEHP: Di-(2-ethylhexyl) phthalate
PAEs: Phthalic acid esters
BBP: Benzyl butyl phthalate
DBP: Di-n-butyl phthalate
PVC: Polyvinylchloride
GC–MS: Gas chromatography mass spectrometry
DMP: Di-methyl phthalate
DEP: Di-ethyl phthalate
MSM: Mineral salts medium
MEHP: Monoethylhexyl phthalate
PA: Phthalic acid
2-EH: 2-Ethylhexanol
