# Supplementary material for: Efficient biodegradation of di-(2-ethylhexyl) phthalate by a novel strain Nocardia asteroides LMB-7 isolated from electronic waste soil
Source: Sci Rep. 2022 Sep 10;12:15262. doi: 10.1038/s41598-022-19752-x (PMC9464244; doi:10.1038/s41598-022-19752-x)
Supplement: Supplementary file 1 — Supplementary Information. [file 41598_2022_19752_MOESM1_ESM.pdf]

Efficient biodegradation of Di-(2-ethylhexyl) Phthalate by a novel strain *Nocardia asteroides* LMB-7 isolated from electronic waste soil

Tian-Tian Chang, Zhi-Wei Lin, Liu-Qing Zhang, Wei-Bing Liu*, Ying Zhou*, Bang-Ce Ye

Lab of Biosystems and Microanalysis, State Key Laboratory of Bioreactor Engi neering, East China University of Science and Technology, Meilong RD 130, S hanghai, 200237, China;

***Corresponding authors:**

E-mail: lwb@ecust.edu.cn, zhouying@ecust.edu.cn

Phone: (+) 00862164253832

Fax: (+) 00862164252094

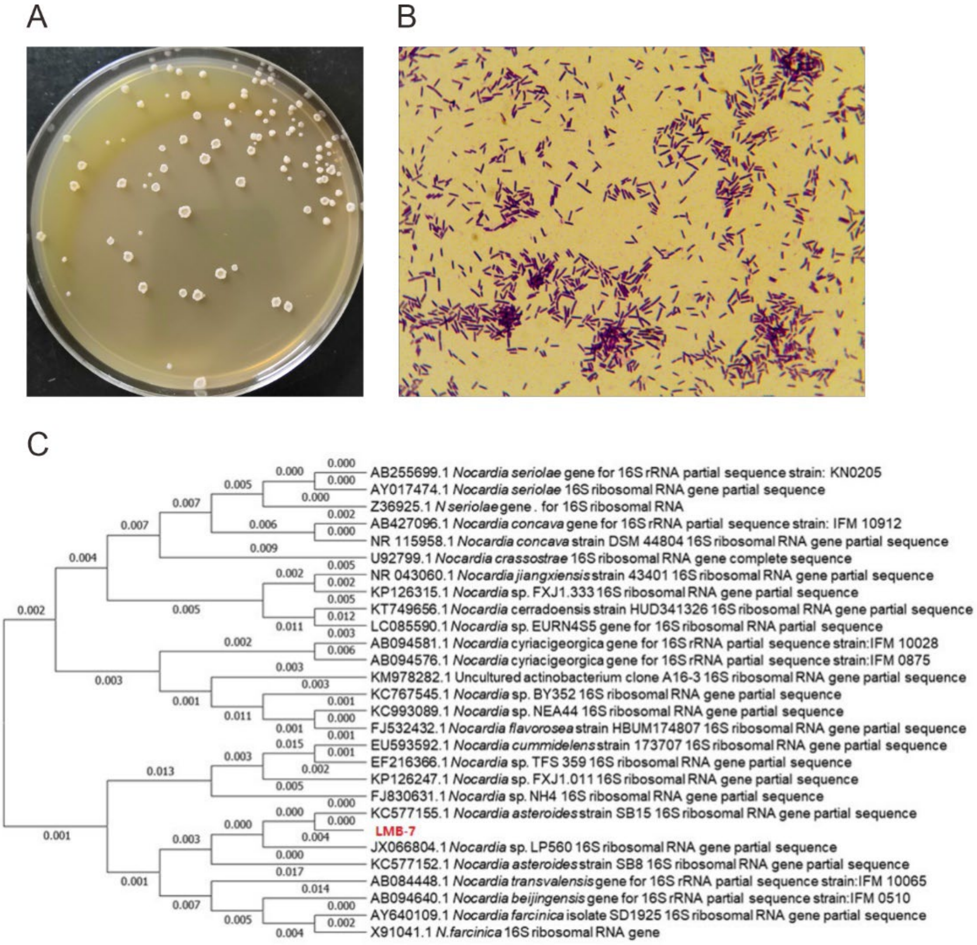

Fig. S1 (A)Colonies, (B) gram stain of LMB-7 strain, and (C) phylogenetic analysis tree with 16s rRNA sequence.

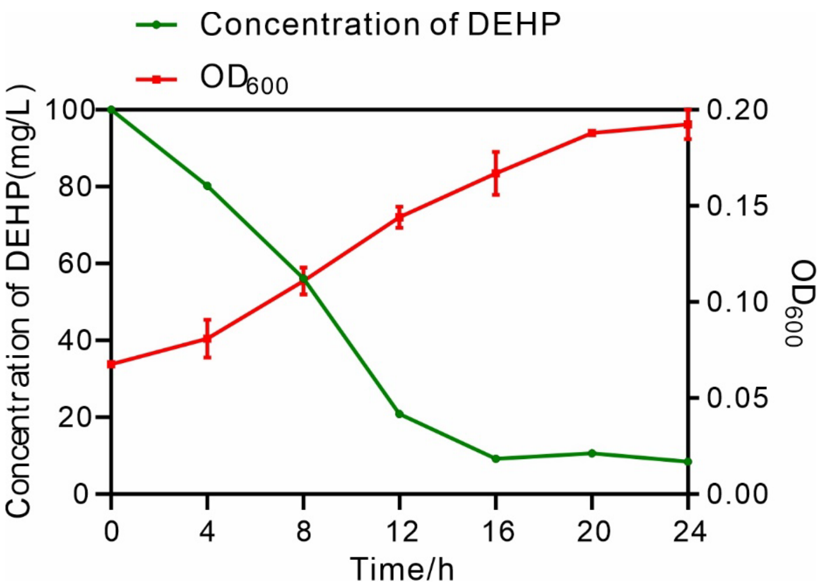

Fig. S2 Correlation of cell growth of LMB-7 and DEHP removal.

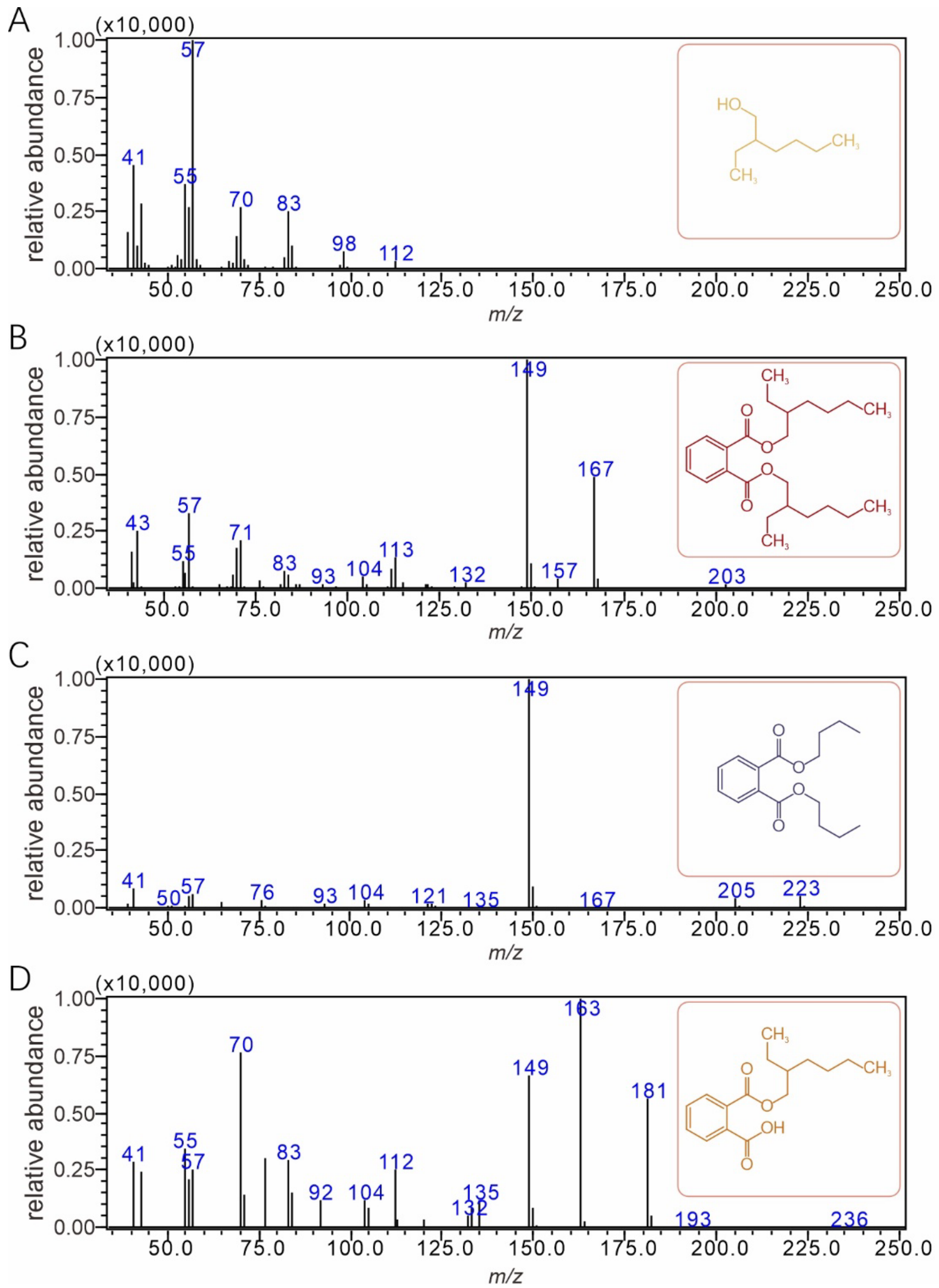

Fig. S3 Intermediate metabolites in the pathway of DEHP biodegradation were identified by GC-MS with A. 2-EH; B. DEHP; C. DBP; D. MEHP
